# Neural Correlates of Duration Discrimination in Young Adults with Autism Spectrum Disorder, Attention-Deficit/Hyperactivity Disorder and Their Comorbid Presentation

**DOI:** 10.3389/fpsyt.2018.00569

**Published:** 2018-11-14

**Authors:** Steve D. Lukito, Owen G. O'Daly, David J. Lythgoe, Susannah Whitwell, Amanda Debnam, Clodagh M. Murphy, Karen Ashwood, Vladimira Stoencheva, Emily Simonoff, Katya Rubia

**Affiliations:** ^1^Department of Child and Adolescent Psychiatry, Institute of Psychiatry, Psychology and Neuroscience, King's College London, London, United Kingdom; ^2^Department of Neuroimaging, Institute of Psychiatry, Psychology and Neuroscience, King's College London, London, United Kingdom; ^3^The Adult Attention-Deficit Hyperactivity Disorder National Service, Behavioural and Developmental Psychiatry Clinical Academic Group, South London and Maudsley NHS Foundation Trust, London, United Kingdom; ^4^Behavioural Genetics Clinic, Adult Autism Service, Behavioural and Developmental Psychiatry Clinical Academic Group, South London and Maudsley NHS Foundation Trust, London, United Kingdom; ^5^Department of Forensic and Neurodevelopmental Sciences, Sackler Institute for Translational Neurodevelopmental Sciences, Institute of Psychiatry, Psychology and Neuroscience, King's College London, London, United Kingdom

**Keywords:** duration discrimination, neurodevelopment disorder, attention-deficit/hyperactivity disorder (ADHD), autism spectrum disorder (ASD), functional magnetic resonance imaging, comorbidity, time estimation

## Abstract

Attention-deficit/hyperactivity disorder (ADHD) and autism spectrum disorder (ASD) often co-occur and share neurocognitive deficits. One such shared impairment is in duration discrimination. However, no studies using functional magnetic resonance imaging (fMRI) have investigated whether these duration discrimination deficits are underpinned by the same or different underlying neurofunctional processes. In this study, we used fMRI to compare the neurofunctional correlates of duration discrimination between young adult males with ASD (*n* = 23), ADHD (*n* = 25), the comorbid condition of ASD+ADHD (*n* = 24), and typical development (TD, *n* = 26) using both region of interest (ROI) and whole brain analyses. Both the ROI and the whole-brain analyses showed that the comorbid ASD+ADHD group compared to controls, and for the ROI analysis relative to the other patient groups, had significant under-activation in right inferior frontal cortex (IFG) a key region for duration discrimination that is typically under-activated in boys with ADHD. The findings show that in young adult males with pure ASD, pure ADHD and comorbid ASD+ADHD with no intellectual disability, only the comorbid group demonstrates neurofunctional deficits in a typical duration discrimination region.

## Introduction

Autism spectrum disorder (ASD) is characterized by difficulties in reciprocal social communication/interaction and stereotyped and repetitive behaviors, while attention-deficit/hyperactivity disorder (ADHD) is characterized by age-inappropriate symptoms of inattention, hyperactivity and impulsivity (1). Both conditions co-occur despite their distinctive diagnostic criteria even in adulthood [e.g., (2, 3)]. Consequently, studies have compared the cognitive and neural correlates of these conditions, and their comorbid presentation (i.e., ASD+ADHD), with the intention of predicting a model of impairments for the comorbid group in relation to the “pure” disorders (4–6).

Perceptual timing, i.e., “the ability to estimate explicitly attended temporal intervals” (7), could be used for investigating potentially different underlying substrates of the pure disorders and of the comorbid disorder. People with both psychiatric conditions are often impaired in daily functions involving timing and time perception skills, including planning and organizing (8–11). Impaired timing is a major pathway to ADHD (7, 12–14) and symptoms of impulsivity, such as verbal blurting and aversion toward delays, have been found to be correlated with impaired time perception (14–16). Anecdotal accounts from parents and clinicians also suggested timing problems in individuals with ASD (17, 18), which possibly forms the basis for checking behavior or strict adherence to routine (18, 19). Furthermore, time perception difficulties are associated with executive function (EF) deficits [e.g., (20–22)], which have been found to be present in these related conditions (23, 24).

Experimental studies of time perception have consistently reported impaired task performance in ADHD [see (7, 14, 25)]. Consistently, reports show that children and adults with ADHD are less able to detect changes in time intervals within the millisecond range [e.g., (26–28)] and tend to over-estimate supra-second time intervals relative to typically developing controls (12, 29–31). Such difficulties, according to reviews and meta-analyses of fMRI studies in people with ADHD, are related to functional impairments in inferior frontal, inferior parietal, striatal, and cerebellar regions (7, 32, 33). Furthermore, during discrimination of intervals differing by several hundreds of milliseconds specifically, ADHD boys have shown under-activation in right dorsolateral prefrontal (DLPFC), bilateral inferior frontal gyrus (IFG), dorsal anterior cingulate/supplementary motor area (DACC/SMA), striatum, left inferior parietal lobe (IPL) and left cerebellum (14, 34–38).

Experimental findings have also suggested an impairment of time perception in people with ASD (39–44) although there are also some negative results (45–47). Despite evidence for time estimation deficits in ASD, no studies have tested the neural substrates of these deficits or compared patients with ADHD and ASD in time perception.

Several studies, however, have compared the two disorders in other EF domains [e.g., (4, 48, 49)], which have been shown to have some overlap in neural activations with time perception (50). During motor inhibition, ASD-specific over-activation was found in bilateral IFG while ADHD-specific under-activation was observed in ventrolateral prefrontal cortex and basal ganglia (49). Furthermore, shared under-activation of the DLPFC was found in both disorders during sustained attention and working memory tasks (4, 48). Interestingly, a study of temporal discounting (6), which is closely related to timing functions (14, 51, 52), showed weaker brain-behavior association in the ASD+ADHD group in typical areas of temporal discounting such as ventromedial and lateral prefrontal cortex, ventral striatum, and anterior cingulate, indicating increased severity of neural impairments in the comorbid than the pure disorders.

To address the gap in the literature, this study explores the neural correlates of time discrimination in young adult males with ASD, ADHD, and ASD+ADHD relative to age-matched controls. We were particularly interested in the potential impairments in the comorbid group compared to the pure groups to elucidate the mechanisms underpinning the co-occurrence of ASD and ADHD, as this information could be useful clinically for formulating disorder-specific treatments. Based on previous fMRI studies of time perception in ADHD, we hypothesized functional impairments in regions previously implicated with timing deficits in the ADHD group, i.e., in ACC/SMA, IFG, caudate, IPL and cerebellum (14, 34–38), with similar but more pronounced deficits in these timing regions in the comorbid group (6), but potentially different abnormalities in the ASD group, based on more inconsistent findings of time perception deficits in ASD [e.g., (41, 44)].

## Methods and materials

### Participants

Participants were 107 young adults aged 20–27 years with ASD, ADHD, ASD+ADHD, and TD and full-scale intelligent quotient (FSIQ) ≥ 70, estimated using the Wechsler Abbreviated Scale of Intelligence-II (WASI-II) (53). Only males were included to increase homogeneity since ASD and ADHD are highly prevalent among males (54, 55). There were equal proportions of left- and right-handed participants across groups, assessed on the Edinburgh Handedness Inventory (56). Excluded were individuals with epilepsy, personality disorder, current substance abuse/dependence, or lifetime history of bipolar disorder, schizophrenia or head injury. Participants in the clinical groups were invited from adult ASD and ADHD clinics, support organizations, social media and an ASD epidemiological cohort the Special Needs and Autism Project (SNAP) (57). Prescriptions of psychostimulants or selective serotonin reuptake inhibitors (SSRIs) were not exclusion criteria for the clinical groups, but psychostimulants were withdrawn 48 h prior to the study. Participants completed an investigation involving several fMRI tasks and a neurocognitive task battery. For the present study, nine subjects were excluded [seven due to excessive motion (>3 mm), one due to missing behavioral data caused by technical issues and another due to incidental MRI finding]. The final sample comprised 23 ASD, 25 ADHD, 24 ASD+ADHD, and 26 TD subjects (Table 1).

**Table 1:** Group differences in socio-demographic variables and clinical measures.

	**TD (*****n*** = **26)**		**ASD (*****n*** = **23)**		**ADHD (*****n*** = **25)**		**ASD**+**ADHD (*****n*** = **24)**		**Group comparison**			***Post-hoc***
	**M**	**SD**	**M**	**SD**	**M**	**SD**	**M**	**SD**	***F/t***	***df***	***p***	
Age	23.4	1.5	23.0	0.7	23.1	1.9	22.9	1.3	0.46	3, 94	0.71	--
FSIQ	117.3	12.0	103.7	18.4	116.0	13.2	106.9	15.9	4.9	3, 94	0.003	ADHD, TD > ASD^*^
Handedness	66.2	69.3	68.3	63.8	65.2	66.1	51.9	71.9	0.29	3, 94	0.83	--
**CAARS ADHD index (*****t*****-scores)**												
Self-rated	41.8	8.5	44.6	11.7	65.2	7.7	59.1	11.8	31.3	3, 94	<0.001	ADHD, ASD+ADHD > ASD^***^; TD^***^
Informant-rated	--	--	48.8	7.2	60.4	16.5	64.5	17.4	7.3	2, 69	<0.001	ADHD, ASD+ADHD > ASD^*^
**SDQ17**+ **Hyperactivity/Inattention (raw scores)**												
Self-rated	2.4	1.8	3.4	2.2	7.4	1.5	6.9	2.1	42.8	3, 86	<0.001	ADHD, ASD+ADHD > ASD^***^, TD^***^
Informant-rated	--	--	3.1	1.9	7.4	2.0	7.1	1.7	38.1	2, 66	<0.001	ASD+ADHD, ADHD > ASD^***^
**ADHD symptom counts^(a)^**												
Inattention	--	--	--	--	8.2	1.3	7.5	1.1	−1.5	1, 40	0.13	--
Hyperactivity/impulsivity	--	--	--	--	5.1	2.6	4.6	2.8	−1.4	1, 40	0.68	--
**Total SRS-2 (*t*-scores)**												
Self-rated	48.5	6.1	61.3	8.9	62.7	6.9	66.7	12.2	20.6	3, 93	<0.001	ASD, ADHD, ASD+ADHD > TD^***^
Informant-rated	--	--	63.8	8.6	56.9	10.5	69.9	11.6	9.4	2, 67	<0.001	ASD+ADHD > ADHD^***^; ASD > ADHD^*^
**ADOS-2 Module 4^(b)^**												
Communication	--	--	1.8	2.0	--	--	2.1	2.3	−0.44	1, 37	0.66	--
Social interaction	--	--	3.3	2.7	--	--	4.0	3.9	−0.65	1, 37	0.52	--
Communication + social interaction	--	--	5.4	4.1	--	--	6.1	6.0	−0.61	1, 37	0.55	--
Stereotyped behaviors and restricted interest	--	--	0.3	0.9	--	--	1.0	1.3	−1.9	1, 37	0.07	--

*TD, Typical development; ASD, Autism Spectrum Disorder; ADHD, Attention Deficit and Hyperactivity Disorder; M, mean; SD, standard deviation; FSIQ, full-scale intelligence quotient; CAARS, Conners Adult ADHD Rating Scale; SRS-2, Social Responsiveness Scale version 2; SDQ17+, Strengths and Difficulties Questionnaires for adults*.

(a) *Current ADHD symptom counts were based on the Diagnostic Interview for Adult ADHD (DIVA 2.0) or the Young Adult Psychiatric Assessment (YAPA), available in 18 participants with ADHD and 16 participants with ASD+ADHD*.

(b) Current ADOS-2 scores were available in a subset of 18 individuals with ASD and 14 participants with ASD+ADHD. Post-hoc significant threshold:

* *p < 0.05*,

*** *p < 0.001*.

In the ASD group, 15 participants had clinical diagnoses confirmed by consultant psychiatrists specialized in ASD (eight with autism; seven with Asperger's syndrome) and eight had research diagnoses of ASD through the SNAP study [three with autism; four with atypical autism, one with pervasive developmental disorder (PDD) unspecified], according to the International Classification of Diseases (ICD-10) criteria (58). Twenty-two ASD diagnoses were supported by gold-standard research instruments, the Autism Diagnostic Observation Schedule (ADOS) (59); three were accompanied by parent interviews on the Autism Diagnostic Interview-Revised (ADI-R) (60). Ten participants met the current cut-off criteria for ASD on at least one of these measures. One participant without ADOS or ADI-R report received childhood ASD diagnosis from a consultant psychiatrist in a specialist neurodevelopmental clinic supported by an assessment on the Diagnostic Interview for Social and Communication Disorders (DISCO) (61) but had no current scores.

All pure ADHD participants met the current DSM5 diagnostic criteria for ADHD, fifteen with combined, nine with predominantly inattentive and one with predominantly hyperactive presentation, diagnosed by consultant psychiatrists in specialist adult ADHD clinics. Twenty-two diagnoses were supported by the Diagnostic Interview for Adult ADHD (DIVA 2.0) (62) and three by the Conners' Adult ADHD Diagnostic Interview for DSM-IV (CAADID) or other type of psychiatric interviews (no current scores) (63). Symptoms of ADHD are reported on Table 2. Five participants were prescribed psychostimulants (four methylphenidate [MPH], one lisdexamphetamine), two SSRIs (sertraline, escitalopram) and one both (MPH, sertraline).

**Table 2:** Behavioral measures of the duration discrimination task across groups.

	**TD (*n* = 26)**	**ASD (*n* = 23)**	**ADHD (*n* = 25)**	**ASD+ADHD (*n* = 24)**
% Mean error DD (SD)	21.7 (10.6)	23.3 (15.7)	23.0 (14.2)	28.0 (13.4)
% Mean error TOJ (SD)	15.3 (12.7)	18.7 (6.0)	19.0 (13.3)	17.3 (14.3)
Mean RT DD (SD)	591.3 (115.3)	560.1 (175.3)	618.1 (159.3)	572.3 (135.5)
Mean RT TOJ (SD)	426.4 (91.4)	402.4 (118.5)	437.5 (146.4)	427.4 (106.9)
SDRT DD (SD)	203.9 (72.7)	192.3 (90.0)	220.6 (86.7)	224.9 (101.9)
SDRT TOJ (SD)	141.3 (74.2)	122.4 (63.2)	158.5 (80.2)	183.2 (91.2)

*Comparison of measures during the duration discrimination task indicated no difference across groups in performance in accuracy, MRT, and SDRT. The MRT and SDRT are in seconds, whereas accuracy is presented as raw number where the maximum was 30. MRT, Mean response time; SDRT, standard deviation of response time, a measure of response time variability and SD, standard deviation*.

In the ASD+ADHD group, eighteen participants had clinical ASD diagnoses (five with autism; eleven with Asperger's syndrome; two with atypical autism) and six had research diagnoses through SNAP [four with atypical autism, two with PDD unspecified], based on the ICD-10 criteria. Twenty-one diagnoses were supported by the ADOS and/or the ADI-R. Eleven met the current cut-off criteria for ASD on at least one of these measures. Three without ADOS or ADI-R reports received childhood ASD diagnoses from consultant psychiatrists in a specialist neurodevelopmental clinic supported by the DISCO (no current scores). Fifteen had clinical DSM5 ADHD diagnoses, 11 of which were supported by current scores of DIVA 2.0, four clinical diagnoses were supported assessment on the CAADID or other psychiatric interview methods (no current scores). Nine participants had a significant history of ADHD symptoms assessed through SNAP (three of whom had clinical diagnosis of ADHD) and met current ADHD DSM5 criteria on the Young Adult Psychiatric Assessment (YAPA) (64). Sixteen met the criteria for combined and eight for inattentive DSM5 ADHD subtype. Six were prescribed psychostimulant (five MPH, one dexamphetamine), two SSRIs (sertraline, escitalopram) and one both (MPH, sertraline).

The TD participants were from local communities, had no psychiatric disorders, were medication-free and scored below clinical cut-off for ADHD and ASD traits on the Conners' Adult ADHD Rating Scale (CAARS) (65) and the Social Responsiveness Scale-2 (SRS-2) (66). This study was in accordance of the Declaration of Helsinki and received ethical approval from a local National Health Service Research Ethics Committee (13/LO/0373). Each participant gave written informed consent and was given £50 for their time.

### Clinical measures

ADHD traits were measured using the ADHD index on the CAARS and the hyperactive/impulsive and inattention domain on the Strengths and Difficulties Questionnaires for adults (SDQ17+; provided by Professor Robert Goodman at the IoPPN). Autistic traits were indexed using the total SRS-2 score. All participants completed self-report measures, corroborated by informants (parents/partner/siblings) for those in the clinical groups.

### Time discrimination task

**Figure 1:**
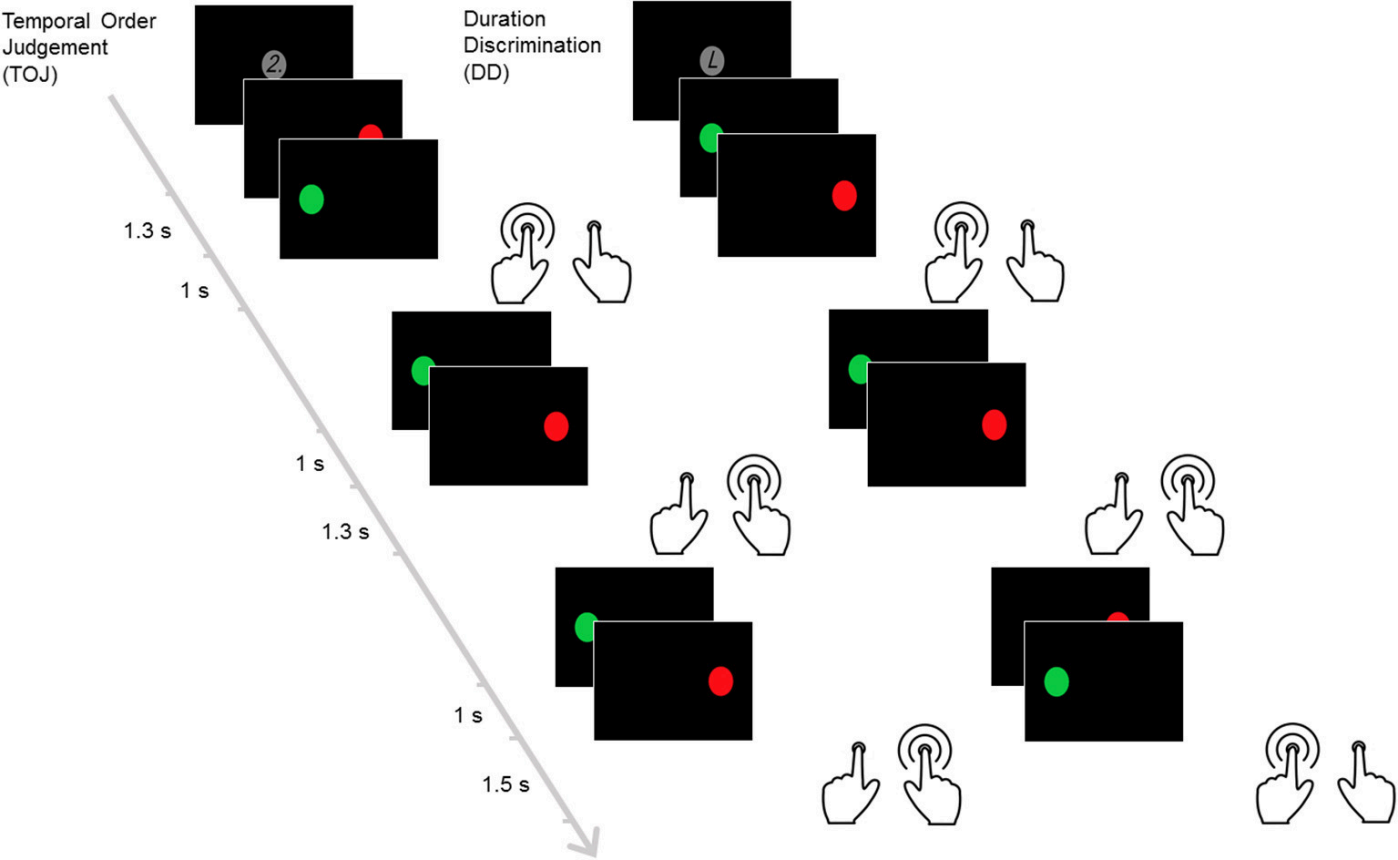
The time discrimination task. This figure illustrates the temporal order judgement and duration discrimination blocks within the task. In this task, pairs of circles (red and green) are presented sequentially. Each temporal order judgment block begins with a screen showing the number “2.” In this block, participants are required to identify the circle that appears at later time between the pairs. Each duration discrimination block starts after the letter “L” is displayed instead. The participants are required to identify the circle that appears for longer duration between the pairs.

This block-design time discrimination task (14, 34–37, 67) consisted of ten 30-s blocks, alternating between duration discrimination (DD) and temporal order judgment (TOJ) blocks. During the DD block, the participants indicated which circle stayed for a longer time on the screen. In the TOJ block, the participants indicated the circle that was shown second. Each block began with a 3-s cue. The cue “2” signaled the start of the TOJ block while “L” signaled the DD block (Figure 1). Each block consisted of six trials. In each trial, a pair of circles (green and red) appeared sequentially on the left- and right-hand side of the screen and equal number of trials started from the left and the right side first. Each block consisted of two trials comparing a 1,000-ms standard interval against a 1,300-, 1,400-, or 1,500-ms test duration, followed by a 2100-ms response period. Participants responded as soon as the second circle was presented. Discrimination errors were the primary outcome measures while mean response time (MRT) and standard deviation of response time (SDRT) were secondary measures. Higher values in these variables reflect increased impairments.

### fMRI data acquisition and analyses

Neuroimaging data were acquired on a General Electric MR750 3T scanner (Boston, MA, USA) at King's College London. The scanner's body coil was used for RF transmission while an 8-channel head coil was used for signal reception. The echo planar image (EPI) gradient-echo pulse sequence (TR/TE = 2,000/30 ms, flip angle = 75°, FOV = 21 × 21 cm, 64 × 64 matrix, in-plane resolution = 3 mm, slice thickness/gap = 3.0/0.3 mm) was used to acquire 41 consecutive, top-to-bottom, slices of T2^*^-weighted MR images parallel to the inter-commissural plane covering the entire brain. The 5-min scan produced 153 volumes in time series. A whole-brain high resolution structural T1-weighted scan (Sagittal ADNI Go/2 ACC SPGR) was acquired in the inter-commissural plane with TR/TE = 7.312/3.016 s, 196 slices, FOV = 27 × 27 cm, 256 × 256 matrix and slice thickness of 1.2 mm.

Each participant's echo-planar imaging (EPI) data were slice-time corrected, realigned, co-registered to the individual's structural T1-weighted scan, segmented, normalized to the Montreal Neurological Institute (MNI) EPI template and smoothed with an 8-mm Gaussian kernel. Statistical analyses were completed in two steps on the Statistical Parametric Mapping (SPM8). At the subject-level, BOLD response was predicted using a vector of onsets and durations convolved with the canonical hemodynamic response function. Six nuisance motion regressors (x-, y-, z-translations and rotations) and separate regressors for each spike (>1 mm) controlled the effects of volume-to-volume head motion and abrupt movements. A high-pass filter (128 s) was applied and a first-order autoregressive model corrected the time series correlation. The contrast DD > TOJ indexed neural correlates of time processing.

Within-group activations (reported in Supplement) were analyzed at the second-level analysis with a cluster extent threshold of *p* < 0.05, family-wise error corrected (FWE_cor_) and a voxel threshold of *p* < 0.001. Between-group activations were analyzed using univariate ANCOVA with group as independent factor, covarying total frame-wise head displacement, in spherical region of interests (ROIs, 10-mm radius) associated with perceptual timing in the general population, drawn from a meta-analysis (68), in the bilateral IFG, SMA, left putamen, left pre-central gyrus, right middle temporal gyrus, right DLPFC, right cingulate gyrus, left insula, and left supramarginal gyrus. The influence of IQ alone on the findings was investigated by adding FSIQ as covariate. To examine the influence of current medication alone, medication status (0 = medicated or 1 = non-medicated) was added in the initial model as covariate. The influence of both medication status and IQ on the group difference findings was assessed by adding both factors as covariates. Additionally, a sensitivity analysis was carried out in participants with no psychotropic medication (22 TD, 21 ASD, 18 ADHD, and 17 ASD+ADHD). BOLD activations were extracted from the clusters using the MarsBaR toolbox (69) for *post-hoc* pairwise comparisons and further correlational analyses. Finally, to explore neural impairments across groups that are non-specific to ADHD, we conducted a whole-brain ANOVA analysis with Group as predictor and whole-brain *t*-contrasts between each clinical group against the TD controls (cluster extent threshold of *p* < 0.05 FWE_cor_ and voxel threshold of *p* < 0.001).

### Statistical analyses

Phenotypic, behavioral, and extracted BOLD data were analyzed using IBM SPSS Statistics 22 (IBM Corp., 2013). Demographic data and phenotypic reports were analyzed using univariate ANOVAs. Task performance measures (Errors, MRT, and SDRT) were analyzed using a 4 × 2 mixed-design ANOVA (Group × Condition), prior which error rates were square-rooted to normalize their distribution *post-hoc* pairwise multiple comparisons were corrected with the Tukey-Kramer method. Correlations between BOLD activations and task performance or phenotypic traits were conducted per group.

## Results

### Participant characteristics

Groups did not differ in age or handedness, but in FSIQ [*F*_(3, 94)_ = 4.9, *p* = 0.003], which was higher in the ADHD (*p* = 0.012), TD groups (*p* = 0.033) and, at trend level, the ASD+ADHD (*p* = 0.089) relative to the ASD group. Groups differed in self-rated ADHD index [*F*_(3, 94)_ = 31.3, *p* < 0.001], the SDQ score [*F*_(3, 94)_ = 42.8, *p* < 0.001] and the informant-rated scores for these measures [*F*_(3, 94)_ = 7.3–38.1, *p*s < 0.001], with *post-hoc* t-tests indicating higher ADHD symptoms in the ADHD and ASD+ADHD groups than the TD (*p*s < 0.001) and the ASD groups (*p*s < 0.001) according to the young adults, which was corroborated by informant-ratings. Self-reported autistic traits were higher in all clinical groups [*F*_(3, 94)_ = 20.6, *p* < 0.001] than controls (all *p*s < 0.001), although informant-rated ASD traits [*F*_(3, 94)_ = 9.4, *p* < 0.001) were significantly higher in the ASD+ADHD (*p* < 0.001) and, at trend-level, in the ASD (*p* = 0.064) relative to the ADHD group.

### Performance results

Errors, MRT and SDRT were greater during DD than TOJ [*F*s_(1, 94)_ ≥ 26.1, *p*s < 0.001] but no main Group effect [*F*s_(3, 94)_ ≤ 1.90, *p*s ≥ 0.14] or Group × Condition interaction effect [*F*s_(3, 94)_ ≤ 0.67, *p*s ≥ 0.67] were observed.

### Neuroimaging results

#### Motion

Group difference in the total volume-to-volume head movement in the x-, y-, and z- rotation and translation was significant [*F*_(3, 94)_ = 2.65, *p* = 0.05] and was covaried in the second-level analysis.

#### Within-group brain activations

**Figure 2:**
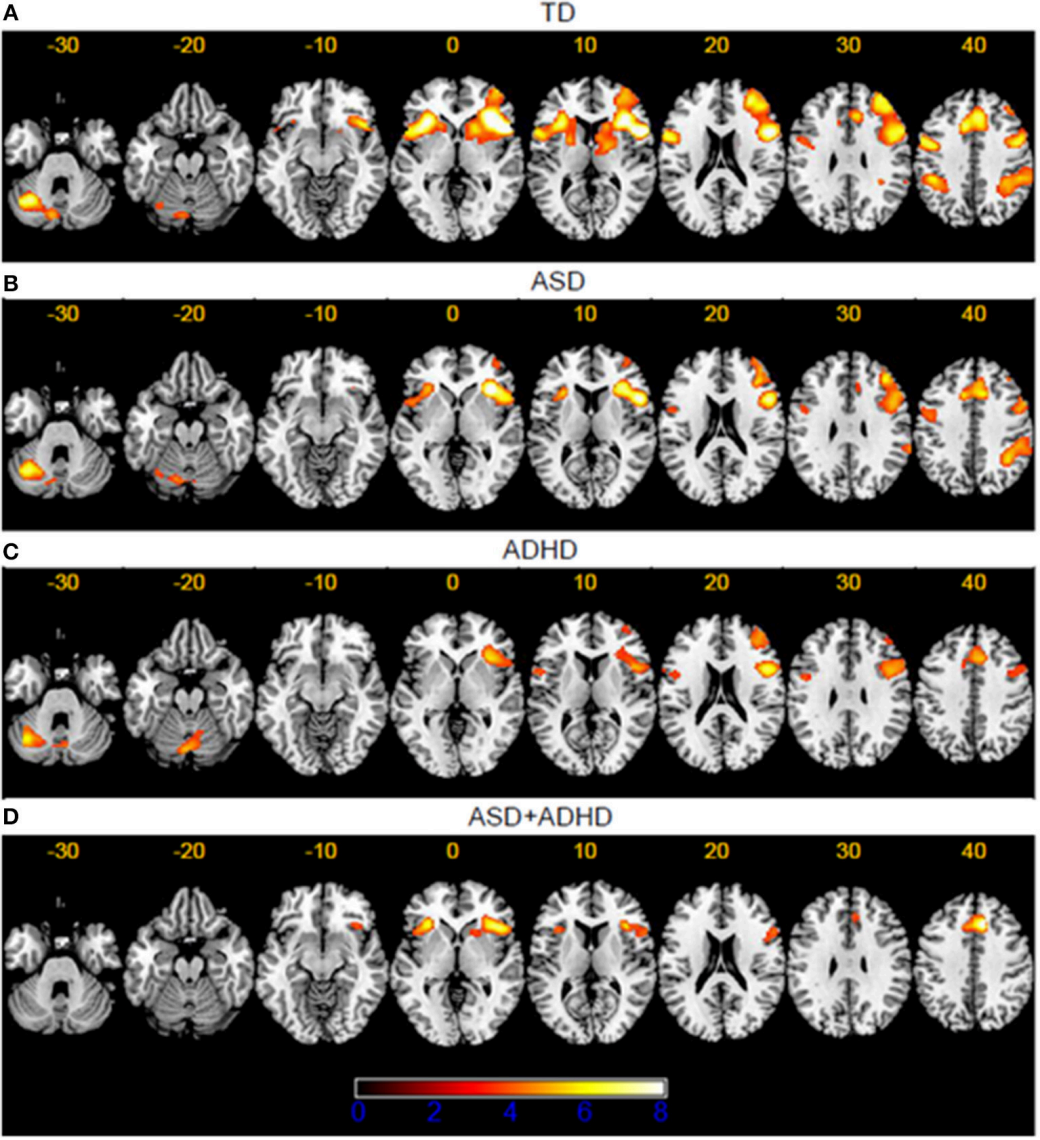
Within-group brain activation clusters contrasting the block duration discrimination vs. temporal order judgement in the **(A)** TD, **(B)** ASD, **(C)** ADHD, and **(D)** ASD+ADHD groups.

The TD group showed activation in right IFC/anterior insula (AI; BA47/13/44/45/46), reaching into the striatum/thalamus; frontally to the dlPFC (BA10/9) and pre-central gyrus (pre-CG; BA6) and medially to the mid-cingulate gyrus/mPFC/SMA (BA32). Activations were also observed in left IFC/AI (BA47/13), reaching into striatum/pallidum; in bilateral IPL/supramarginal/angular gyri (BA40) and left posterior cerebellum. In ASD, clusters of activations were less extensive than in TD although mostly overlapping, including right IFC/AI (BA44/45/46/13), dlPFC (BA6), mid-cingulate gyrus/SMA (BA32), and premotor and superior frontal gyrus (SFG; BA10). Also included were left IFC/AI (BA45/13), right IPL/supramarginal/angular gyri (BA40), and left cerebellum, and left pre-CG (BA6). Participants with ADHD showed smaller clusters than the TD and ASD groups in right IFC/dlPFC (BA44/45/46/9/8) reaching into pre-CG (BA6), in left posterior cerebellum extending to right cerebellar lobe, in cingulate gyrus reaching to mPFC/SMA areas (BA24/32/6), and in left IFC/pre-CG (BA44/45/6). The clusters in the ASD+ADHD group were the least extensive and found in mPFC/SMA (BA24/32/6), bilateral AI/IFC (BA13/47/45), reaching into caudate/putamen on the right hemisphere (see Figure 2).

#### Between-group brain activations

**Figure 3:**
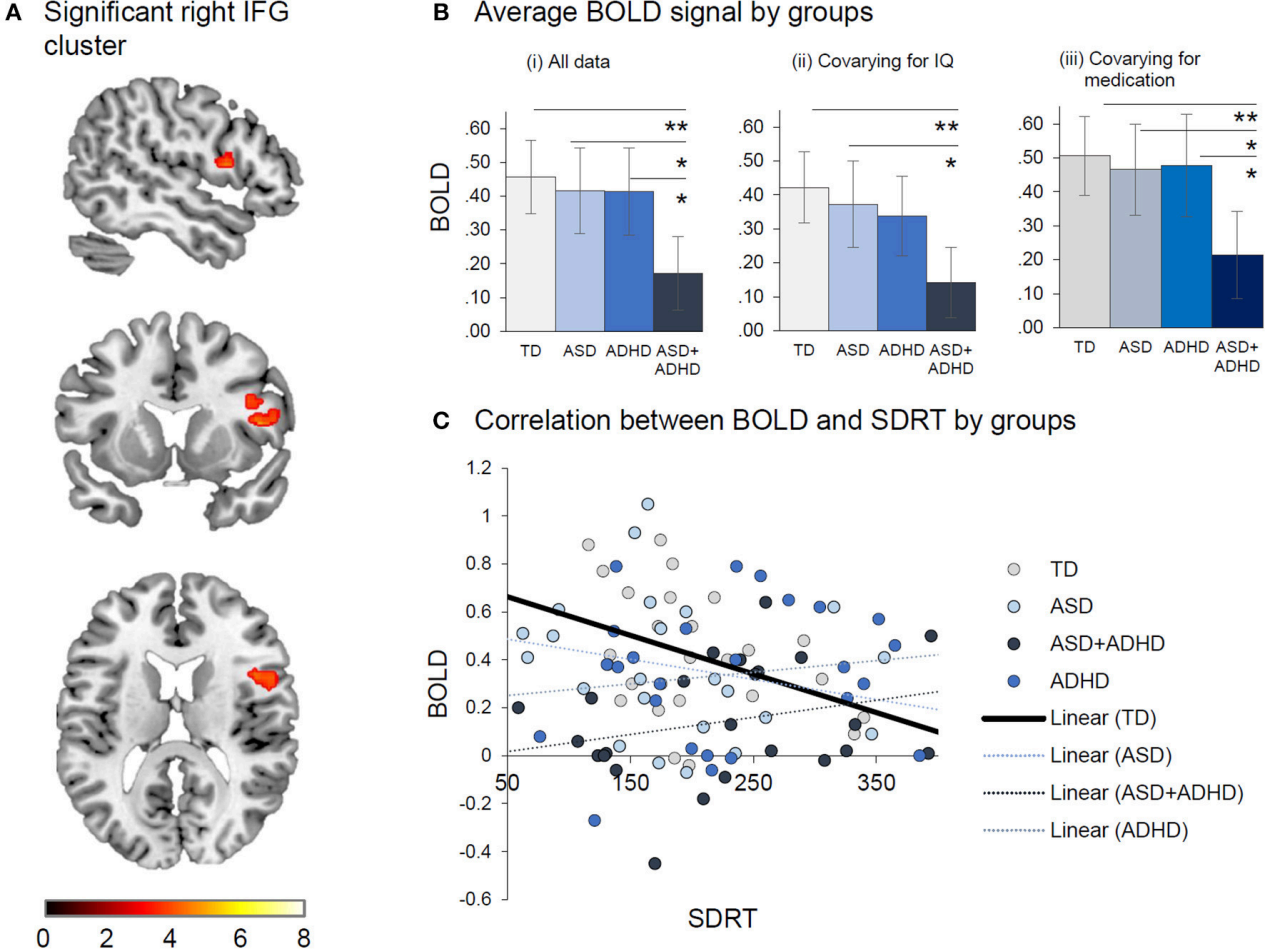
Between-group effects of duration discrimination vs. temporal order judgement. **(A)** Significant right IFG cluster where Group effect was found. **(B)** Average BOLD signal by group is displayed (i) for all data, (ii) after covarying for IQ and (iii) covarying for medication. **(C)** Correlation between BOLD and SDRT by group indicates that reduced BOLD is associated with increased in SDRT. *p < 0.05, **p < 0.01.

In the ROIs typically activated during perceptual timing, a group effect was observed in a right IFG cluster [*p* = 0.049, *F* = 5.5, (10, 12, 46), *k*_E_ = 56 voxels] (see Figure 3), with *post-hoc* comparisons showing that the ASD+ADHD group had less activation than the other groups (*p*s ≤ 0.024). Covarying for IQ reduced the group effect to a trend level [*p* = 0.09, *F* = 4.8, (10, 12, 46), *k*_E_ = 18 voxels], preserving the pairwise difference between the ASD+ADHD and the TD or ASD (*p*s ≤ 0.027), but not the ADHD group (*p* = 0.08). Group effect findings in right IFG cluster were maintained when medication status alone [*p* = 0.019, *F* = 6.5, (10, 12, 48), *k*_E_ = 235 voxels], and when both medication status and IQ were covaried [*p* = 0.039, *F* = 5.8, (10, 12, 48), *k*_E_ = 137 voxels] and the *post-hoc* pairwise analyses consistently showed reduced activation in the ASD+ADHD relative to other groups (*p*s ≤ 0.040) in this cluster. Sensitivity analyses excluding participants on medication showed the same group effect [*p* = 0.036, *F* = 6.0, (10, 12, 52), *k*_E_ = 49 voxels]. However, *post-hoc t*-tests showed reduced activation in the ASD+ADHD group relative to the TD and ASD (*p*s ≤ 0.008) but not the ADHD group (*p* = 0.51). No other significant pairwise differences were observed. The right IFG activation cluster correlated negatively with SDRT in the TD [*r*_TD_ (26) = −0.43; *p* = 0.03]; but not the clinical groups (*|rs*| ≤ 0.18; *p* ≥ 0.38).

Whole-brain analyses revealed under-activation in right IFG/DLPFC cluster in the ASD+ADHD relative to the TD group [*p* = 0.033, *t* = 4.2, (8, 32, 36), *k*_E_ = 440], which was preserved when medication status was covaried [*p* = 0.002, *t* = 4.5, (10, 10, 48), *k*_E_ = 864 voxels] but did not survive after covarying for IQ, after covarying for both medication status and IQ, or in the sensitivity analysis excluding those who were prescribed medication. No other comparisons between the clinical and TD groups yielded significant differences.

## Discussion

The study was aimed at elucidating the similarities and differences in the neural correlates of duration discrimination in young adult males with ASD, ADHD, and ASD+ADHD. The groups had comparable task performance. However, people with ASD+ADHD had under-activation in right IFG relative to the clinical and TD groups in ROIs most consistently activated during time perception (68). In support of this finding, under-activation in right IFG/DLPFC was found only in the ASD+ADHD group relative to TD controls in the exploratory whole-brain analyses. This suggests that in adulthood, only people with ASD+ADHD, but not the pure disorders are impaired in the key region that mediates time discrimination.

The lack of neurofunctional impairment in the ADHD group is not in line with the hypothesized under-activation in right IFG based on reports in adolescents with ADHD during the same task (14, 33–38), which could have several possible explanations. First, previous reports have examined adolescents rather than adults with ADHD. Thus, it is possible that adults with ADHD no longer demonstrate the lateral frontal functional deficits related to time perception observed at younger ages, as has also been shown during response inhibition in adults with ADHD compared to typically developing adults in some previous studies (70–72). Second, compared to previous studies of timing (14, 34–38), the ADHD participants in this study had above-average IQ, which might have moderated time-processing related neural activation deficits, since covarying for IQ reduced the statistical significance of the difference in right IFG activation between the ADHD and the ASD+ADHD group to trend level. Third and most importantly, the lack of neurofunctional abnormalities in the ADHD relative to the TD group could be related to current psychotropic medication prescription in the sample. A sensitivity analysis excluding participants with psychotropic medication (mostly psychostimulants) revealed that the difference between the ASD+ADHD and ADHD groups was no longer significant, suggesting a subtle subthreshold abnormality that may still have been present in the non-medicated ADHD group. This is in line with the typical observation of right IFG under-activation during duration discrimination in medication-naïve ADHD children (14, 34–36), and the findings of an association between single-dose and long-term psychostimulant administration with the upregulation and normalization of right IFG under-activation during timing and other tasks in ADHD children (14, 35, 36, 73–76). This interpretation should, however, be taken with caution as an analysis covarying for medication retained the right IFG under-activation finding in the ASD+ADHD relative to the ASD, ADHD, and TD, which did not differ from one another, suggesting that the exclusion of medicated participants during the sensitivity analyses may have led to reduced power.

The lack of neural impairments in the ASD group was not in line with the initial hypothesis, which, in the absence of fMRI studies of timing in ASD, was formulated based on behavioral findings only [e.g., (39, 42)]. However, negative neurobehavioral findings from some studies (45–47), including a recent study in adults with ASD (77), have suggested heterogeneity in timing impairments in this population. Therefore, although the implication of the present neurofunctional finding is that timing networks in young adults with ASD without intellectual disability are unimpaired, this must be taken mindful of factors that could have increased the participants' heterogeneity in the study. First, participants in the ASD and ASD+ADHD group in this study had varying presentations (autism, Aspergers, atypical autism, PDD unspecified), which could be associated with heterogeneous neural impairments [see (78, 79)]. Second, a sizeable number of people with ASD in this study are community-sampled and may have less severe impairment than individuals who are clinically referred (80). This is supported by the fact that among those who had current ADOS or ADI-R, only approximately half the participants in the ASD and the ASD+ADHD groups met the clinical cut-off criteria on either measure. Time perception network abnormalities may thus be present in clinically referred children and adults with ASD. Another consideration is the known relationship between time perception and a number of other EF domains such as inhibition (20, 22), working memory and sustained attention (81–83). Meta-analytic findings suggest that these different cognitive functions are subserved by overlapping regions (50). It is hence possible that poor behavioral performance during time perception tasks found in boys and adult males with ASD in previous studies was mediated by a variety of individually specific deficits in neural networks for other EF domains (4, 6, 48, 49) rather than those subserving time perception *per se*.

The novel finding of right IFG under-activation in the ASD+ADHD group during DD in the ROI and the whole-brain analyses indicate that the addition of ADHD symptoms in adults with ASD lead to increased neurofunctional impairments during DD. This interpretation extends, in the domain of time perception, a previous finding by Chantiluke et al. (6) based on weaker brain-behavior associations in the comorbid group relative to the pure groups in brain regions associated with temporal reward discounting. Since adults with ASD+ADHD had similar neural impairments as ADHD boys, which are no longer observed in the ADHD adult group, the findings could also indicate more persistent impairments in the comorbid relative to the pure ADHD group. This hypothesis could be further explored by replicating the study involving participants with a wider age range including adolescents and adults.

Analyses of behavioral measures suggest comparable task performance across all participant groups, which was unexpected in people with ADHD where duration discrimination deficits have been reported [e.g., (7, 26–28)]. However, tasks typically lose behavioral sensitivity when adapted for fMRI studies (84, 85), and the recommended sample size for fMRI studies of over 20 (86) may not be sufficiently powered for detecting group differences in behavioral performance on fMRI tasks. In fact, not all findings of neural impairments were accompanied by task performance deficits in ADHD children compared to healthy controls in previous studies of duration discrimination (37, 38). However, a brain-behavior association between increased right IFG activation and reduced response time variability (SDRT) was found in the typically developing group, underlining the importance of this region for task performance in the healthy population. Increased intra-subject SDRT is an indicator of attentional lapses during cognitive tasks (87, 88) which, during DD, disturbs the perception of the passage of time (89). Thus, increased activation in right IFG in the control group may also reflect better attentional control during time perception.

A strength of the study is the robust characterization of diagnoses of ASD and ADHD in the majority of patients, including the use of ICD-10, DSM5, ADI-R, ADOS, DIVA 2.0, and CAADID, as appropriate (although current scores were not available for everyone). One weakness was the IQ difference across groups which resulted in altered findings of the exploratory whole-brain analysis when IQ was covaried. However, the use of ANCOVA to correct for IQ when it is intrinsically different between groups [e.g., between individuals with neurodevelopmental disorders relative to TD controls (90–92)] and thus when the group memberships are not randomly assigned, is statistically not appropriate since it can lead to artifactual positive or negative results (93–95). Furthermore, covarying for IQ mostly preserved the conclusion from the ROI analyses especially with respect to the under-activation of right IFG in the ASD+ADHD group relative to other groups, suggesting that the study was not entirely powered for exploratory whole-brain analyses. Another limitation is the inclusion of people currently prescribed medication in the ADHD and ASD+ADHD groups as both SSRIs and stimulants have been shown to affect brain activation in people with these conditions [e.g., (35, 96, 97)]. However, findings remained when we covaried for medication status and sensitivity analyses excluding individuals on medication did not change the primary finding between the ASD+ADHD and TD groups in this study. Additionally, the narrow age range (20–27 years) of the young adult participants limits generalizability of findings to the entire adult ADHD and ASD populations. Finally, fMRI has limitations with respect to temporal resolution. While we were mainly interested in the spatial location of shared or disorder-specific brain abnormalities in relation to the process of temporal judgement in the disorders, adding a temporally better resolved method would have allowed us to also understand differences between disorders in the exact time course of activation deficits. Thus, future studies may consider the combined use of fMRI and physiological (e.g., electroencephalography or magnetoencephalography) approaches with high temporal resolution to complement the fMRI findings during time estimation.

## Conclusions

In summary, only young adult males with comorbid ASD+ADHD showed reduced activation in right IFG during duration discrimination relative to healthy controls and the pure groups who were unimpaired. The findings suggest that right IFG is not neurofunctionally impaired in young adults with ADHD or ASD during time perception, although the finding in the ADHD group particularly has to be viewed in light of the possible moderating influence of IQ and medication use among the participants in this study.

## Author contributions

SL, KR, and ES conceptualized the study and contributed to manuscript preparation. SL conducted the recruitment and data collection. SL and OO analyzed the data. DL contributed to the manuscript preparation. SW, AD, CM, KA, and VS conducted recruitment and contributed to the manuscript preparation.

### Conflict of interest statement

KR has received grants from Lilly and Shire pharmaceuticals and speaker's bureau from Shire, Lilly and Medice. The remaining authors declare that the research was conducted in the absence of any commercial or financial relationships that could be construed as a potential conflict of interest.

## References

[1] American Psychiatric Association (APA) Diagnostic and Statistical Manual of Mental Disorders: DSM-5. Washington, DC (2013).

[2] Joshi G Wozniak J Petty C Martelon MK Fried R Bolfek A. Psychiatric comorbidity and functioning in a clinically referred population of adults with autism spectrum disorders: A comparative study. J Autism Dev Disord. (2013) 43:1314–25. 10.1007/s10803-012-1679-5 23076506

[3] Johnston K Dittner A Bramham J Murphy C Knight A Russell A. Attention deficit hyperactivity disorder symptoms in adults with autism spectrum disorders. Autism Res. (2013) 6:225–36. 10.1002/aur.1283 23788522

[4] Christakou A Murphy CM Chantiluke K Cubillo AI Smith AB Giampietro V. Disorder-specific functional abnormalities during sustained attention in youth with attention deficit hyperactivity disorder (ADHD) and with autism. Mol Psychiatry (2013) 18:236–44. 10.1038/mp.2011.185 22290121 PMC3554878

[5] Di Martino A Zuo XN Kelly C Grzadzinski R Mennes M Schvarcz A. Shared and distinct intrinsic functional network centrality in autism and attention-deficit/hyperactivity disorder. Biol Psychiatry (2013) 74:623–632. 10.1016/j.biopsych.2013.02.011 23541632 PMC4508007

[6] Chantiluke K Christakou A Murphy CM Giampietro V Daly EM Ecker C. Disorder-specific functional abnormalities during temporal discounting in youth with attention deficit hyperactivity disorder (ADHD), autism and comorbid ADHD and autism. Psychiatry Res. (2014) 223:113–20. 10.1016/j.pscychresns.2014.04.006 24929553

[7] Noreika V Falter CM Rubia K. Timing deficits in attention-deficit/hyperactivity disorder (ADHD): evidence from neurocognitive and neuroimaging studies. Neuropsychologia (2013) 51:235–66. 10.1016/j.neuropsychologia.2012.09.036 23022430

[8] Boyer BE Geurts HM Van der Oord S. Planning skills of adolescents with ADHD. J Atten Disord. (2014) 22:46–57. 10.1177/1087054714538658 24972795

[9] Kofler MJ Sarver DE Harmon SL Moltisanti A Aduen PA Soto EF. Working memory and organizational skills problems in ADHD. J Child Psychol Psychiatry (2018) 59:57–67. 10.1111/jcpp.12773 28714075 PMC5729117

[10] Kenworthy LE Black DO Wallace GL Ahluvalia T Wagner AE Sirian LM. Disorganization: the forgotten executive dysfunction in high-functioning autism (HFA) spectrum disorders. Dev Neuropsychol. (2005) 28:809–27. 10.1207/s15326942dn2803_4 16266250

[11] van den Bergh SF Scheeren AM Begeer S Koot HM Geurts HM. Age related differences of executive functioning problems in everyday life of children and adolescents in the autism spectrum. J Autism Dev Disord. (2014) 44:1959–71. 10.1007/s10803-014-2071-4 24562693

[12] Barkley RA Murphy KR Bush T. Time perception and reproduction in young adults with attention deficit hyperactivity disorder. Neuropsychology (2001) 15:351–60. 10.1037/0894-4105.15.3.351 11499990

[13] de Zeeuw P Weusten J van Dijk S van Belle J Durston S. Deficits in cognitive control, timing and reward sensitivity appear to be dissociable in ADHD. PLoS ONE (2012) 7:e51416. 10.1371/journal.pone.0051416 23236497 PMC3517570

[14] Rubia K Halari R Christakou A Taylor E. Impulsiveness as a timing disturbance: neurocognitive abnormalities in attention-deficit hyperactivity disorder during temporal processes and normalization with methylphenidate. Philos Trans R Soc Lond B Biol Sci. (2009) 364:1919–31. 10.1098/rstb.2009.0014 19487194 PMC2685816

[15] Baumann AA Odum AL. Impulsivity, risk taking, and timing. Behav Process. (2012) 90:408–14. 10.1016/j.beproc.2012.04.005 22542458 PMC3897391

[16] Wittmann M Simmons AN Flagan T Lane SD Wackermann J Paulus MP. Neural substrates of time perception and impulsivity. Brain Res. (2011) 1406:43–58. 10.1016/j.brainres.2011.06.048 21763642 PMC3145046

[17] Hewetson A The Stolen Child: Aspects of Autism and Asperger Syndrome. Westport, CT; London: Bergin & Garvey (2002).

[18] Wing L The Autistic Spectrum: A Guide for Parents and Professionals. London: Constable (1996)

[19] Peeters T Gillberg C Autism: Medical and Educational Aspects. London: Whurr (1999).

[20] Brown SW Perreault ST. Relation between temporal perception and inhibitory control in the Go/No-Go task. Acta Psychol. (2017) 173:87–93. 10.1016/j.actpsy.2016.12.004 28024254

[21] Grinblat N Rosenblum S. Why are they late? Timing abilities and executive control among students with learning disabilities. Res Dev Disabil. (2016) 59:105–114. 10.1016/j.ridd.2016.07.012 27525557

[22] Ogden RS Salominaite E Jones LA Fisk JE Montgomery C. The role of executive functions in human prospective interval timing. Acta Psychol. (2011) 137:352–8. 10.1016/j.actpsy.2011.04.004 21561595

[23] Craig F Margari F Legrottaglie AR Palumbi R de Giambattista C Margari L. A review of executive function deficits in autism spectrum disorder and attention-deficit/hyperactivity disorder. Neuropsychiatr Dis Treat. (2016) 12:1191–202. 10.2147/NDT.S104620 27274255 PMC4869784

[24] Rommelse NNJ Geurts HM Franke B Buitelaar JK Hartman CA. A review on cognitive and brain endophenotypes that may be common in autism spectrum disorder and attention-deficit/hyperactivity disorder and facilitate the search for pleiotropic genes. Neurosci Biobehav Rev. (2011) 35:1363–96. 10.1016/j.neubiorev.2011.02.015 21382410

[25] Falter CM Noreika V Time processing in developmental disorders: a comparative view. In: Arstila V Lloyd D, editors, Subjective Time. Cambridge, MA: MIT Press (2014). pp. 557–598.

[26] Smith AB Taylor E Rogers JW Newman S Rubia K. Evidence for a pure time perception deficit in children with ADHD. J Child Psychol Psychiatry (2002) 43:529–42. 10.1111/1469-7610.00043 12030598

[27] Toplak M Tannock R. Time perception: modality and duration effects in attention-deficit/hyperactivity disorder (ADHD). J Abnorm Child Psychol. (2005) 33:639–54. 10.1007/s10802-005-6743-6 16195956

[28] Valko L Schneider G Doehnert M Müller U Brandeis D Steinhausen H-C. Time processing in children and adults with ADHD. J Neural Transm. (2010) 117:1213–28. 10.1007/s00702-010-0473-9 20821338

[29] Barkley RA Edwards G Laneri M Fletcher K Metevia L. Executive functioning, temporal discounting, and sense of time in adolescents with attention deficit hyperactivity disorder (ADHD) and oppositional defiant disorder (ODD). J Abnorm Child Psychol. (2001) 29:541–56. 10.1023/A:1012233310098 11761287

[30] Bauermeister JJ Barkley RA Martinez JV Cumba E Ramirez RR Reina G. Time estimation and performance on reproduction tasks in subtypes of children with attention deficit hyperactivity disorder. J Clin Child Adolesc Psychol. (2005) 34:151–62. 10.1207/s15374424jccp3401_14 15677289

[31] McGee R Brodeur D Symons D Andrade B Fahie C. Time perception: does it distinguish ADHD and RD children in a clinical sample? J Abnorm Child Psychol. (2004) 32:481–490. 10.1023/B:JACP.0000037778.61929 15500028

[32] Droit-Volet S. Time perception in children: a neurodevelopmental approach. Neuropsychologia (2013) 51:220–34. 10.1016/j.neuropsychologia.2012.09.023 22999968

[33] Hart H Radua J Mataix-Cols D Rubia K. Meta-analysis of fMRI studies of timing in attention-deficit hyperactivity disorder (ADHD). Neurosci Biobehav Rev. (2012) 36:2248–56. 10.1016/j.neubiorev.2012.08.003 22922163

[34] Hart H Marquand AF Smith A Cubillo A Simmons A Brammer M. Predictive neurofunctional markers of attention-deficit/hyperactivity disorder based on pattern classification of temporal processing. J Am Acad Child Adolesc Psychiatry (2014) 53:569–78.e1. 10.1016/j.jaac.2013.12.024 24745956

[35] Rubia K Alegria AA Cubillo AI Smith AB Brammer MJ Radua J. Effects of stimulants on brain function in attention-deficit/hyperactivity disorder: a systematic review and meta-analysis. Biol Psychiatry (2014) 76:616–28. 10.1016/j.biopsych.2013.10.016 24314347 PMC4183380

[36] Smith AB Cubillo A Barrett N Giampietro V Simmons A Brammer M. Neurofunctional effects of methylphenidate and atomoxetine in boys with attention-deficit/hyperactivity disorder during time discrimination. Biol Psychiatry (2013) 74:615–22. 10.1016/j.biopsych.2013.03.030 23731741

[37] Smith AB Taylor E Brammer M Halari R Rubia K. Reduced activation in right lateral prefrontal cortex and anterior cingulate gyrus in medication-naive adolescents with attention deficit hyperactivity disorder during time discrimination. J Child Psychol Psychiatry (2008) 49:977–85. 10.1111/j.1469-7610.2008.01870.x 18759938

[38] Vloet TD Gilsbach S Neufang S Fink GR Herpertz-Dahlmann B Konrad K. Neural mechanisms of interference control and time discrimination in attention-deficit/hyperactivity disorder. J Am Acad Child Adolesc Psychiatry (2010) 49:356–67. 10.1097/00004583-201004000-00010 20410728

[39] Allman MJ DeLeon IG Wearden JH. Psychophysical assessment of timing in individuals with autism. Am J Intellect Dev Disabil. (2011) 116:165–78. 10.1352/1944-7558-116.2.165 21381951 PMC4822529

[40] Baron-Cohen S Wheelwright S Scahill V Lawson J Spong A Are intuitive physics and intuitive psychology independent? A test with children with Asperger syndrome. J Dev Learn Disord. (2001) 5:47–78.

[41] Falter CM Noreika V Wearden JH Bailey AJ. More consistent, yet less sensitive: interval timing in autism spectrum disorders. Q J Exp Psychol. (2012) 65:2093–107. 10.1080/17470218.2012.690770 22800511

[42] Kargas N López B Reddy V Morris P. The relationship between auditory processing and restricted, repetitive behaviors in adults with autism spectrum disorders. J Autism Dev Disord. (2015) 45:658–68. 10.1007/s10803-014-2219-2 25178987

[43] Maister L Plaisted-Grant KC Time perception and its relationship to memory in autism spectrum conditions: time perception in ASC. Dev Sci. (2011) 14:1311–22. 10.1111/j.1467-7687.2011.01077.x 22010891

[44] Lepisto T Silokallio S Nieminen-von Wendt T Alku P Naatanen R Kujala T. Auditory perception and attention as reflected by the brain event-related potentials in children with Asperger syndrome. Clin Neurophysiol. (2006) 117:2161–71. 10.1016/j.clinph.2006.06.709 16890012

[45] Jones CR Happe F Baird G Simonoff E Marsden AJ Tregay J. Auditory discrimination and auditory sensory behaviours in autism spectrum disorders. Neuropsychologia (2009) 47:2850–8. 10.1016/j.neuropsychologia.2009.06.015 19545576

[46] Kofler MJ Rapport MD Bolden J Sarver DE Raiker JS. ADHD and working memory: the impact of central executive deficits and exceeding storage/rehearsal capacity on observed inattentive behavior. J Abnorm Child Psychol. (2010) 38:149–61. 10.1007/s10802-009-9357-6 19787447

[47] Wallace GL Happe F Time perception in autism spectrum disorders. Res Autism Spectr Disord. (2008) 2:447–55. 10.1016/j.rasd.2007.09.005

[48] Chantiluke K Barrett N Giampietro V Brammer M Simmons A Rubia K. Disorder-dissociated effects of fluoxetine on brain function of working memory in attention deficit hyperactivity disorder and autism spectrum disorder. Psychol Med. (2015) 45:1195–205. 10.1017/s0033291714002232 25292351

[49] Chantiluke K Barrett N Giampietro V Santosh P Brammer M Simmons A. Inverse fluoxetine effects on inhibitory brain activation in non-comorbid boys with ADHD and with ASD. Psychopharmacology (2015) 232:2071–82. 10.1007/s00213-014-3837-2 25533997 PMC4432080

[50] Radua J Del Pozo NO Gómez J Guillen-Grima F Ortuño F. Meta-analysis of functional neuroimaging studies indicates that an increase of cognitive difficulty during executive tasks engages brain regions associated with time perception. Neuropsychologia (2014) 58:14–22. 10.1016/j.neuropsychologia.2014.03.016 24709569

[51] Kim BK Zauberman G Perception of anticipatory time in temporal discounting. J Neurosci Psychol Econ. (2009) 2:91–101. 10.1037/a0017686

[52] Jones CR Jahanshahi M. Dopamine modulates striato-frontal functioning during temporal processing. Front Integr Neurosci. (2011) 5:70. 10.3389/fnint.2011.00070 22046150 PMC3200491

[53] Wechsler D Wechsler Abbreviated Scale of Intelligence - 2nd Edition (WASI-II). Bloomington, MN: Pearson (2011).

[54] Fayyad J De Graaf R Kessler R Alonso J Angermeyer M Demyttenaere K. Cross-national prevalence and correlates of adult attention-deficit hyperactivity disorder. Br J Psychiatry (2007) 190:402–9. 10.1192/bjp.bp.106.034389 17470954

[55] Fombonne E. Epidemiology of autistic disorder and other pervasive developmental disorders. J Clin Psychiatry (2005) 66(Suppl. 10):3–8. 16401144

[56] Oldfield RC. The assessment and analysis of handedness: the Edinburgh inventory. Neuropsychologia (1971) 9:97–113. 10.1016/0028-3932(71)90067-4 5146491

[57] Baird G Simonoff E Pickles A Chandler S Loucas T Meldrum D. Prevalence of disorders of the autism spectrum in a population cohort of children in South Thames: the Special Needs and Autism Project (SNAP). Lancet (2006) 368:210–5. 10.1016/S0140-6736(06)69041-7 16844490

[58] World Health Organization (WHO) ICD-10: International Statistical Classification of Diseases and Related Health Problems Tenth Revision. Geneva: WHO (2004). 3376487

[59] Lord C Risi S Lambrecht L Cook EH Jr Leventhal BL DiLavore PC. The Autism Diagnostic Observation Schedule-Generic: a standard measure of social and communication deficits associated with the spectrum of autism. J Autism Dev Disord. (2000) 30:205–23. 10.1023/A:1005592401947 11055457

[60] Lord C Rutter M Le Couteur A. Autism Diagnostic Interview-Revised: a revised version of a diagnostic interview for caregivers of individuals with possible pervasive developmental disorders. J Autism Dev Disord. (1994) 24:659–85. 7814313 10.1007/BF02172145

[61] Wing L Leekam SR Libby SJ Gould J Larcombe M. The Diagnostic Interview for Social and Communication Disorders: background, inter-rater reliability and clinical use. J Child Psychol Psychiatry (2002) 43:307–325. 10.1111/1469-7610.00023 11944874

[62] Kooij JJS Adult ADHD: Diagnostic Assessment and Treatment. Springer: London; New York (2013)

[63] Epstein JN Johnson DE Conners CK Conners' Adult ADHD Diagnostic Interview for DSM-IV. North Tonawanda, NY: Multi-Health Systems Inc (2001).

[64] Angold A Cox A Prendergast M Rutter M Simonoff E The Young Adult Psychiatric Assessment (YAPA) Version 2.0.3. Durham, NC: Duke University (2009).

[65] Conners CK Erhardt D Sparrow E Conners' Adult ADHD Rating Scales (CAARS): Technical Manual. New York, NY; Toronto, ON: Multi-Health Systems Inc (1999).

[66] Constantino JN Gruber C Social Responsiveness Scale™, Second Edition (SRS™-2). Torrance, CA: Western Psychological Services (2012).

[67] Smith A Taylor E Lidzba K Rubia K. A right hemispheric frontocerebellar network for time discrimination of several hundreds of milliseconds. Neuroimage (2003) 20:344–50. 10.1016/S1053-8119(03)00337-9 14527594

[68] Wiener M Turkeltaub P Coslett HB. The image of time: a voxel-wise meta-analysis. Neuroimage (2010) 49:1728–740. 10.1016/j.neuroimage.2009.09.064 19800975

[69] Brett M Anton J-L Valabregue R Poline JB Region of interest analysis using an SPM toolbox. Presented at the 8th International Conference on Functional Mapping of the Human Brain, June 2-6, 2002, Sendai, Japan. Neuroimage (2002) 16(Supplement 1): 1140–1. 10.1016/s1053-8119(02)90013-3

[70] Congdon E Altshuler LL Mumford JA Karlsgodt KH Sabb FW Ventura J. Neural activation during response inhibition in adult attention-deficit/hyperactivity disorder: preliminary findings on the effects of medication and symptom severity. Psychiatry Res Neuroimaging (2014) 222:17–28. 10.1016/j.pscychresns.2014.02.002 24581734 PMC4009011

[71] Carmona S Hoekzema E Antoni Ramos-Quiroga J Richarte V Canals C Bosch R. Response inhibition and reward anticipation in medication-naive adults with attention-deficit/hyperactivity disorder: a within-subject case-control neuroimaging study. Hum Brain Mapp. (2012) 33:2350–61. 10.1002/hbm.21368 21826761 PMC6870239

[72] Chen CY Yen JY Yen CF Chen CS Liu GC Liang CY. Aberrant brain activation of error processing among adults with attention deficit and hyperactivity disorder. Kaohsiung J Med Sci. (2015) 31:179–87. 10.1016/j.kjms.2015.01.001 25835273 PMC11916387

[73] Chou T-L Chia S Shang C-Y Gau SS-F. Differential therapeutic effects of 12-week treatment of atomoxetine and methylphenidate on drug-naive children with attention deficit/hyperactivity disorder: a counting Stroop functional MRI study. Eur Neuropsychopharmacol. (2015) 25:2300–10. 10.1016/j.euroneuro.2015.08.024 26409297

[74] Cubillo A Smith AB Barrett N Giampietro V Brammer MJ Simmons A. Shared and drug-specific effects of atomoxetine and methylphenidate on inhibitory brain dysfunction in medication-naive ADHD boys. Cereb Cortex (2014) 24:174–85. 10.1093/cercor/bhs296 23048018 PMC3862268

[75] Hart H Radua J Nakao T Mataix-Cols D Rubia K. Meta-analysis of functional magnetic resonance imaging studies of inhibition and attention in attention-deficit/hyperactivity disorder: exploring task-specific, stimulant medication, and age effects. JAMA Psychiatry (2013) 70:185–98. 10.1001/jamapsychiatry.2013.277 23247506

[76] Norman LJ Carlisi C Lukito S Hart H Mataix-Cols D Radua J. Structural and functional brain abnormalities in attention-deficit/hyperactivity disorder and obsessive-compulsive disorder: a comparative meta-analysis. JAMA Psychiatry (2016) 73:815–25. 10.1001/jamapsychiatry.2016.0700 27276220

[77] Jones CR Lambrechts A Gaigg SB. Using time perception to explore implicit sensitivity to emotional stimuli in autism spectrum disorder. J Autism Dev Disord. (2017) 47:2054–66. 10.1007/s10803-017-3120-6 28429189 PMC5487748

[78] McAlonan GM Cheung C Cheung V Wong N Suckling J Chua SE. Differential effects on white-matter systems in high-functioning autism and Asperger's syndrome. Psychol Med. (2009) 39:1885–93. 10.1017/s0033291709005728 19356262

[79] McAlonan GM Suckling J Wong N Cheung V Lienenkaemper N Cheung C. Distinct patterns of grey matter abnormality in high-functioning autism and Asperger's syndrome. J Child Psychol Psychiatry (2008) 49:1287–95. 10.1111/j.1469-7610.2008.01933.x 18673405

[80] Goodman SH Lahey BB Fielding B Dulcan M Narrow W Regier D. Representativeness of clinical samples of youths with mental disorders: a preliminary population-based study. J Abnorm Psychol. (1997) 106:3–14. 9103713 10.1037//0021-843x.106.1.3

[81] Lee HY Yang EL. Exploring the effects of working memory on time perception in attention deficit hyperactivity disorder. Psychol Rep. (2018). 10.1177/0033294118755674. [Epub ahead of print]. 29417882

[82] Pan Y Luo QY. Working memory modulates the perception of time. Psychon Bull Rev. (2012) 19:46–51. 10.3758/s13423-011-0188-4 22124848

[83] Polti I Martin B van Wassenhove V. The effect of attention and working memory on the estimation of elapsed time. Sci Rep. (2018) 8:6690. 10.1038/s41598-018-25119-y 29703928 PMC5923266

[84] Rubia K Smith AB Brammer MJ Toone B Taylor E. Abnormal brain activation during inhibition and error detection in medication-naive adolescents with ADHD. Am J Psychiatry (2005) 162:1067–75. 10.1176/appi.ajp.162.6.1067 15930054

[85] Smith AB Taylor E Brammer M Toone B Rubia K. Task-specific hypoactivation in prefrontal and temporoparietal brain regions during motor inhibition and task switching in medication-naive children and adolescents with attention deficit hyperactivity disorder. Am J Psychiatry (2006) 163:1044–51. 10.1176/ajp.2006.163.6.1044 16741205

[86] Thirion B Pinel P Mériaux S Roche A Dehaene S Poline JB. Analysis of a large fMRI cohort: statistical and methodological issues for group analyses. Neuroimage (2007) 35:105–20. 10.1016/j.neuroimage.2006.11.054 17239619

[87] Huang-Pollock CL Karalunas SL Tam H Moore AN. Evaluating vigilance deficits in ADHD: a meta-analysis of CPT performance. J Abnorm Psychol. (2012) 121:360–71. 10.1037/a0027205 22428793 PMC3664643

[88] Haynes BI Bauermeister S Bunce D. Does within-person variability predict errors in healthy adults aged 18-90? Q J Exp Psychol. (2017) 70:1722–31. 10.1080/17470218.2016.1204328 27328052

[89] Zakay D. Relative and absolute duration judgments under prospective and retrospective paradigms. Percept Psychophys. (1993) 54:656–64. 8290334 10.3758/bf03211789

[90] Charman T Pickles A Simonoff E Chandler S Loucas T Baird G. IQ in children with autism spectrum disorders: data from the Special Needs and Autism Project (SNAP). Psychol Med. (2011) 41:619–27. 10.1017/s0033291710000991 21272389

[91] Postorino V Fatta LM Sanges V Giovagnoli G De Peppo L Vicari S. Intellectual disability in autism spectrum disorder: investigation of prevalence in an Italian sample of children and adolescents. Res Dev Disabil. (2016) 48:193–201. 10.1016/j.ridd.2015.10.020 26619372

[92] Rommel AS Rijsdijk F Greven CU Asherson P Kuntsi J. A longitudinal twin study of the direction of effects between ADHD symptoms and IQ. PLoS ONE (2015) 10:e0124357. 10.1371/journal.pone.0124357 25875897 PMC4398424

[93] Evans SH Anastasio EJ. Misuse of analysis of covariance when treatment effect and covariate are confounded. Psychol Bull. (1968) 69:225–34. 10.1037/h0025666 5659655

[94] Dennis M Francis DJ Cirino PT Schachar R Barnes MA Why IQ is not a covariate in cognitive studies of neurodevelopmental disorders. J Int Neuropsychol Soc. (2009) 15:331 10.1017/s1355617709090481 19402919 PMC3075072

[95] Miller GM Chapman JP. (2001) Misunderstanding analysis of covariance. J Abnorm Psychol. (2012) 110:40–8. 10.1037/0021-843x.110.1.40 11261398

[96] Chantiluke K Barrett N Giampietro V Brammer M Simmons A Murphy DG. Inverse effect of fluoxetine on medial prefrontal cortex activation during reward reversal in ADHD and autism. Cereb Cortex (2015) 25:1757–70. 10.1093/cercor/bht365 24451919 PMC4459282

[97] Murphy DG Daly E Schmitz N Toal F Murphy K Curran S. Cortical serotonin 5-HT2A receptor binding and social communication in adults with Asperger's syndrome: an *in vivo* SPECT study. Am J Psychiatry (2006) 163:934–6. 10.1176/ajp.2006.163.5.934 16648340

